# Exogenous Application of 5-Aminolevulinic Acid Promotes Coloration and Improves the Quality of Tomato Fruit by Regulating Carotenoid Metabolism

**DOI:** 10.3389/fpls.2021.683868

**Published:** 2021-06-09

**Authors:** Junwen Wang, Jing Zhang, Jing Li, Mohammed Mujitaba Dawuda, Basharat Ali, Yue Wu, Jihua Yu, Zhongqi Tang, Jian Lyu, Xuemei Xiao, Linli Hu, Jianming Xie

**Affiliations:** ^1^College of Horticulture, Gansu Agricultural University, Lanzhou, China; ^2^Department of Horticulture, FoA, University for Development Studies, Tamale, Ghana; ^3^Department of Agronomy, University of Agriculture, Faisalabad, Pakistan; ^4^Gansu Provincial Key Laboratory of Aridland Crop Science, Lanzhou, China

**Keywords:** 5-aminolevulinic acid, tomato fruit, internal quality, coloration, lycopene synthesis

## Abstract

5-Aminolevulinic acid (ALA) plays an important role in plant growth and development. It can also be used to enhance crop resistance to environmental stresses and improve the color and internal quality of fruits. However, there are limited reports regarding the effects of ALA on tomato fruit color and its regulatory mechanisms. Therefore, in this study, the effects of exogenous ALA on the quality and coloration of tomato fruits were examined. Tomato (*Solanum lycopersicum* “Yuanwei No. 1”) fruit surfaces were treated with different concentrations of ALA (0, 100, and 200 mg⋅L^–1^) on the 24th day after fruit setting (mature green fruit stage), and the content of soluble sugar, titratable acid, soluble protein, vitamin C, and total free amino acids, as well as amino acid components, intermediates of lycopene synthetic and metabolic pathways, and ALA metabolic pathway derivatives were determined during fruit ripening. The relative expression levels of genes involved in lycopene synthesis and metabolism and those involved in ALA metabolism were also analyzed. The results indicated that exogenous ALA (200 mg⋅L^–1^) increased the contents of soluble sugars, soluble proteins, total free amino acids, and vitamin C as well as 11 kinds of amino acid components in tomato fruits and reduced the content of titratable acids, thus improving the quality of tomato fruits harvested 4 days earlier than those of the control plants. In addition, exogenous ALA markedly improved carotenoid biosynthesis by upregulating the gene expression levels of geranylgeranyl diphosphate synthase, phytoene synthase 1, phytoene desaturase, and lycopene β-cyclase. Furthermore, exogenous ALA inhibited chlorophyll synthesis by downregulating the genes expression levels of Mg-chelatase and protochlorophyllide oxidoreductase. These findings suggest that supplementation with 200 mg⋅L^–1^ ALA not only enhances the nutritional quality and color of the fruit but also promotes early fruit maturation in tomato.

## Introduction

Fruit quality is determined by visual attributes, including size and color, as well as non-visual attributes, such as fruit taste and nutritional value (Nour et al., 2010). In recent years, consumer pursuit of tomato fruit qualities, such as appearance, flavor, and internal nutrition, has increased. Consumers equate the visual appearance of fresh fruits with their internal qualities (Saltveit, 1999), which jointly determines their preferences and purchase desires (Hadi et al., 2013). In fresh fruits, the internal qualities of fruit mainly refer to soluble sugars, organic acids, vitamin C, and other nutrients (Hecke et al., 2006). Moreover, the most important external quality is color, which is one of the critical factors determining consumer acceptance (Yu et al., 2016). Thus, improvement of the external qualities of fruits has become an important research area (Ye et al., 2017).

Tomato (*Solanum lycopersicum*) is one of the most widely produced and consumed fruits worldwide (Zhu et al., 2018). The fruit is rich in nutrients, such as minerals, vitamin C, and lycopene, which are good dietary antioxidants (Perveen et al., 2015; Xie et al., 2019) with the potential to delay aging and inhibit cancer cell proliferation (Buyuklu et al., 2015; Ye et al., 2016). Tomato fruits of different colors contain various pigments, such as carotenoids and anthocyanins (Borghesi et al., 2016). The red, pink, and orange coloration of tomato fruits is mainly attributed to carotenoid accumulation (Yu et al., 2016) and lycopene is the primary carotenoid in ripe tomato fruits (Ip et al., 2013). Lycopene is derived from the 5-carbon compound isopentenyl diphosphate (IPP) in plastids (Hirschberg, 2001). The addition of three IPP molecules to dimethylallyl diphosphate is catalyzed by geranylgeranyl diphosphate synthase (GGPPS) to produce geranylgeranyl diphosphate (GGPP) (Okada et al., 2000). The condensation of two GGPP molecules into phytoene is catalyzed by phytoene synthase (PSY), which is the first step in synthesizing carotenoids in mature tomato fruits (Fraser et al., 2002). Subsequently, phytoene and ζ-carotene undergo desaturation reactions that are catalyzed by phytoene desaturase (PDS) and ζ-carotene desaturase (ZDS) (Fraser et al., 2002). All-*trans-*lycopene is produced by isomerization catalyzed by carotene isomerase (CRTISO); when CRTISO is mutated, the fruit color is orange, and the carotenoids downstream of lycopene are significantly reduced (Isaacson et al., 2002; Fantini et al., 2013).

Multiple strategies for improving fruit coloration have also been reported. For example, lycopene content in tomato fruit can be increased by covering greenhouses with double-layer polyethylene films, thus promoting tomato coloration (Jarquín-Enríquez et al., 2013). Supplementation with red light can stimulate carotenoid accumulation (Dorais et al., 2008). Moreover, use of arbuscular mycorrhizal inoculum of *Funelliformis mosseae* in P-limited soil could improve tomato fruit color and nutritional quality (Palumbo et al., 2020). However, these methods increase the cost of tomato production under greenhouse conditions. Application of plant growth regulators is a convenient and economically viable method for improving plant growth and fruit ripening (Ali et al., 2015; Feng et al., 2016; Xie et al., 2019). For instance, in mango, the climacteric peak and skin coloration are promoted by ethylene application (Mohamed and Abu-Goukh, 2003). Conversely, in tomato fruit, downregulation of key genes in the ethylene biosynthetic pathway, *ACO* or *ACS*, leads to a reduction in lycopene content (Yokotani et al., 2004; Ito et al., 2017). However, exogenous application of ABA promotes carotenoid accumulation and accelerates fruit reddening in mature green tomatoes (Mou et al., 2016). During fruit ripening, brassinolide application effectively induced tomato fruit ripening and increased the lycopene content (Zhu et al., 2015). Among plant growth regulators, 5-aminolevulinic acid (ALA) is an essential biosynthetic precursor of all tetrapyrrole compounds (chlorophyll, heme, and vitamin B_12_) (Senge et al., 2014), which have promotive effects on plant growth and stress resistance, such as seed germination (Korkmaz and Korkmaz, 2009; Fu et al., 2014), plant biomass (Xu et al., 2015), salinity tolerance (Ahmad et al., 2012; Naeem et al., 2012), drought (Li et al., 2011; Liu et al., 2013), and heavy metals (Ali et al., 2013a,b; Gill et al., 2015). In the ALA metabolic pathway, higher-plant chlorophyll has several precursors, including protoporphyrin IX (Proto IX), Mg-protoporphyrin IX (Mg-Proto IX), and protochlorophyllide (Pchlide) (Chen, 2014; Sobotka, 2014; Nguyen et al., 2015).

Several studies have reported that ALA is involved in the regulation of crop nutrient quality and fruit coloration. For example, exogenous application of ALA has been found to increase vitamin C and soluble sugar contents, reduce nitrate and crude fiber contents, and lead to better quality and taste in lettuce (Xu et al., 2012). Application of ALA significantly increased the soluble solid and soluble protein content by 20.9 and 31.4%, respectively, and decreased titratable acid content, thus promote the quality of tomato fruit (Wang et al., 2008). Rhizospheric application of ALA increased contents of ascorbic acid, soluble proteins, soluble solids, and soluble sugars of apple fruit, and decreased titratable acid content, thus improves interior qualities (Zheng et al., 2017). In addition, anthocyanin accumulation was increased in peach skin through the application of exogenous ALA, which upregulated the expression levels of *CHS*, *CHI*, *F3H*, *DFR*, *LDOX*, and *UFGT*, resulting in improved peach coloration (Ye et al., 2017). Another study showed that after ALA treatment, the content of heme (metabolic derivatives of ALA) increased, which may act as a transcription factor, up-regulating the gene expressions of *MYB*, *bHLH*, and *WD40*, the latter in turn up-regulate the expression of the structural genes such as *PAL*, *CHS*, and *UFGT*, thus, anthocyanin was synthesized *de novo* in apple skins (Xie et al., 2013). The expression of *MdMADS1*, a developmental transcription regulator of fruit ripening, was positively correlated with expression of anthocyanin biosynthetic genes (*MdCHS*, *MdDFR*, *MdLDOX*, and *MdUFGT*) in apple skin under ALA treatment, synergistic or additive responses between ALA and *MdMADS1* exists for regulation of apple skin anthocyanin accumulation (Feng et al., 2016). These results indicate that ALA can significantly promote anthocyanin accumulation and fruit coloration. However, the regulative role of ALA on carotenoid pigments biosynthesis, fruit coloration and its mechanisms have not been elucidated yet. Therefore, in the present study, red fruit tomato was used as the experimental material, and the positive effects of exogenous ALA application on the coloration and nutrient quality of tomato fruits during ripening were investigated. Moreover, the relative expression levels of key genes involved in lycopene anabolism were assessed to understand the molecular mechanisms underlying ALA-induced lycopene accumulation.

## Materials and Methods

### Plant Materials and Experimental Design

Tomato (*Solanum lycopersicum* “Yuanwei No. 1”) plants were used in this study and grown in a solar greenhouse in Lanzhou City, China (35.87°N, 104.09°E).

Experiment I, the first fruit clusters (the growth position of the first inflorescence from bottom to top on tomato plants) were used to select the appropriate ALA concentration on fruit coloration and quality. The concentrations of ALA (Sigma-Aldrich, St. Louis, MO, United States) were 0, 50, 100, 200, and 300 mg L^–1^. Tomato fruit were treated at the mature green stage (24 days after fruit setting). Fully expanded fruits with uniform sizes (diameters around 4 cm) were selected and treated at 3-days interval until the fruit matured. These fruit surfaces were evenly daubed with ALA solution containing 0.01% Tween-20. During fruit maturity, the contents of soluble sugar, soluble solids, and fruit firmness were determined to select the appropriate ALA treatment.

Experiment II, the third fruit clusters (the growth position of the third inflorescence from bottom to top on tomato plants) were selected for further test. During experimental treatment, 10 healthy tomato plants with same growth vigor were marked in each treatment, repeated three times. In order to ensure the same fruit maturity of each treatments, 2–3 fruits with the same pollination date were selected as the treatment target on the third fruit clusters. In total, there were about 90 fruits in each treatment. Tomato fruit were treated at the mature green stage (24 days after fruit setting). Fully expanded fruits and fruits of uniform sizes (diameters around 4 cm) were selected and treated at 3-days interval until the fruit matured. The most effective ALA (Sigma-Aldrich, United States) solution containing 0.01% Tween-20 as the surfactant. Three replications were carried out for each treatment, and each replicate included 10 tomato plants. Fruit samples were taken at 3-days interval during the treatment period until the fruits were mature. The tomato fruit was considered mature when 90% of its surface was red. During sampling, five tomato fruits were randomly selected for each treatment, and the treatment was repeated three times. Tomato fruits treated with exogenous ALA and those in the control group matured by the 40th and 44th days after fruit setting, respectively.

### Tomato Fruit Morphology and Skin Color Parameters

After exogenous ALA application, the fruits were photographed at each sampling time point. The skin color of the fruit was measured using a colorimeter (CR-10 Plus, Konica Minolta, Inc., Tokyo, Japan), which provided color surface coordinates *L*^∗^, *a*^∗^, and *b*^∗^ (*L*^∗^ indicates lightness; *a*^∗^ indicates a range between green and red; and *b*^∗^ indicates a range between blue and yellow). The *a*^∗^ and *b*^∗^ values were processed to obtain the hue angle (hue angle value is a comprehensive indicator of color change, which is inversely proportional to *a*^∗^ and directly proportional to *b*^∗^). The hue angle (in degrees) was calculated according to the following equation (Sagar et al., 2013):

$$\mathrm{Hue}=\tan^{-1}\,\left(b^{*}/a^{*}\right),{\mathrm{ifa}}^{*}>0;\mathrm{otherwise},$$

$$\mathrm{Hue}=180+\tan^{-1}\,\left(b^{*}/a^{*}\right),{\mathrm{ifa}}^{*}<0$$

Each sample consisting of three fruits was randomly selected, and measurements were performed at the shoulder, at a point parallel to the equatorial plane, and on the top of each fruit.

### Determination of the Nutrient Quality of Tomato Fruits

The fruit firmness was measured by GY-4-J firmness tester (Top Cloud-agri Technology Co., Ltd., Hangzhou, China). The soluble solids were determined by PAL-1 refractometer (ATAGO Co., Ltd., Japan). Soluble protein content was determined using Coomassie brilliant blue G250 staining (Sedmak and Grossberg, 1977) with some modifications. Fresh tomato fruit samples (0.5 g) were ground, transferred to a centrifuge tube, and deionized water was added to a volume of 10 mL. The extract was centrifuged at 5,000×*g* for 10 min at 4°C, and the supernatant was collected. The soluble protein content was determined at a wavelength of 595 nm. The vitamin C content was determined using an ascorbic acid content detection kit (Solarbio Life Science, Beijing, China) following the manufacturer’s protocol. Total free amino acid content was determined using the ninhydrin assay (Sorrequieta et al., 2010) with some modifications. Briefly, fresh tomato fruit samples (0.25 g) were ground to a fine paste and placed in a volumetric flask. Then, deionized water was added to a volume of 100 mL; the mixture was filtered, and the filtrate was collected. The total free amino acid content was determined at a wavelength of 500 nm.

The soluble sugar content was determined using the method of Grandy et al.’s (2000) with some modifications. Fresh tomato fruit samples (0.2 g) were ground to a fine paste and placed in a sterile test tube to which 5 mL of deionized water was added, and the sample was mixed. This step was repeated twice, and the supernatant was collected after 30 min incubation in a water bath at 100°C. Deionized water was added to a volume of 25 mL. The soluble sugar content was determined using the anthrone method at a wavelength of 620 nm. The titratable acid content of tomato fruits was determined using the method described by Wang et al.’s (2013). Fresh tomato fruit samples (5 g) were ground to a fine paste and placed in an Erlenmeyer flask. Deionized water was then added to a volume of 50 mL, and the mixture was filtered. The filtrate then underwent titration with 0.1 mol⋅L^–1^ sodium hydroxide (containing two drops of 1% phenolphthalein), resulting in a faint pink color, which was used as an indicator. Mean values were calculated from three independent sample replicates.

The sample preparation and LC–MS analysis of amino acid components were according to the method described by Nimbalkar et al.’s (2012) with slight modifications. 0.5 g dried sample was weighed into sample container containing 20 mL of 0.1% (v/v) hydrochloric acid. Ultrasonic extraction for 15 min, followed by centrifugation at 4°C at 10,000 rpm for 5 min. The supernatant was passed through 0.2 μm nylon membrane filter and 5 μL of sample was injected to LC-MS (Agilent 1290-6460, CA, United States) analysis.

### Determination of the Contents of Intermediates of the Lycopene Synthetic and Metabolic Pathways

Carotenoids were isolated according to the method described by Kang et al.’s (2010) with slight modifications. The tomato fruits were dried using a freeze dryer (LyoQuest-85, Telstar Technologies, Barcelona, Spain) and then ground into powder using a grinder (TissueLyser II; QIAGEN, Hilden, Germany). Sample powder (0.5 g) was added to a 30 mL solution (petroleum ether and acetone, 2:1 v/v) and then extracted at 30°C for 40 min by sonication (SB-800 DT, NingBo Scientz Biotechnology Co., Ltd., NingBo, China). After the aqueous phase was removed, the petroleum ether extract was poured into a round-bottom flask, dried by rotary evaporation at 40°C, and then dissolved in 25 mL of a mixture of acetonitrile, dichloromethane, and methanol (55:20:25, v/v/v). The mixture was then filtered through a 0.22 μm organic filter membrane and finally analyzed by high-performance liquid chromatography (HPLC) using a Symmetry C18 column (250 mm × 4.6 mm, 5 μm, Waters Corp., Milford, MA, United States). The flow rate was 1.2 mL⋅min^–1^; the mobile phase was methanol, acetonitrile, and dichloromethane (25:55:20, v/v/v); and the column temperature was maintained at 30°C.

Compounds were detected at 450 nm (β-carotene and lutein), 470 nm (lycopene), and 286 nm (phytoene). The compounds were identified according to the retention times of standards (lycopene, β-carotene, and lutein obtained from YuanYe Biotechnology Co., Ltd., Shanghai, China, and phytoene obtained from Sigma–Aldrich) and quantified according to standard curves. Data were analyzed using Empower Software (Waters Corp.).

### Determination of the Contents of Metabolic Derivatives of the ALA Metabolic Pathway

5-Aminolevulinic acid was determined according to the method described by Wu et al.’s (2018). Briefly, 5 g of fresh tomato fruit sample was ground with 6 mL acetate buffer (pH 4.6) on an ice bath, centrifuged at 5,000×*g* for 15 min at 4°C, and the supernatant collected. The absorbance was measured at 554 nm. The calculation of endogenous ALA concentration is based on the protein concentration of samples as reference (μmol⋅mg^–1^ Prot).

To determine the chlorophyll content of fresh tomato fruit samples, 2 g samples were extracted with 80% buffered aqueous acetone (Porra et al., 1989). The absorbance of the supernatant was determined at 646 and 663 nm. The chlorophyll content (Chl *a* and *b*) was calculated using the following formulas described by Lichtenthaler and Wellburn (1983). *V* values indicate the dissolved volume of the determined solution; FW values indicate the fresh weight of the sample.

$$\text{Chl}\,\text{a}\,\left(\text{mg}\,{\text{g}}^{-1}\,\text{FW}\right)=\left(12.21\times {\text{OD}}_{663}-2.81\times {\text{OD}}_{646}\right)\times V/\text{FW}$$

$$\text{Chl b}\,\left(\mathrm{mg}\,g^{-1}\,\text{FW}\right)=\left(20.13\times {\text{OD}}_{646}-5.03\times {\text{OD}}_{663}\right)\times V/\text{FW}$$

Proto IX, Mg-Proto IX, and Pchlide were determined according to the methods described by Wu et al.’s (2018) with some modifications. Briefly, 1 g of fresh tomato fruit sample was homogenized with 2 mL 80% alkaline acetone, placed in a sterile test tube, and then 80% alkaline acetone was added to a volume of 25 mL. The homogenate was incubated under dark conditions until the sample was bleached. The homogenate was centrifuged at 1,500×*g* for 10 min, and the supernatant was collected. The absorbance of the supernatant was determined at 575, 590, and 628 nm. The results were calculated as described by Liu et al.’s (2015) with some modifications. The calculation of metabolic derivatives concentrations are based on the protein concentration of samples as reference (μmol⋅g^–1^ Prot). V1 values indicate the dissolved volume of the of the sample; V2 values indicate the determined volume of the sample; Cpr values indicate the protein concentration of the sample.

$$\text{Proto}\text{IX}\left(\mu molg^{-1}\text{Prot}\right)=(0.18016\times {\text{OD}}_{575}-0.04036\times {\text{OD}}_{628}-0.0451\times {\text{OD}}_{590})\times \text{V}1/(\text{V}2\times \text{Cpr})$$

$$\text{Mg}-\text{Proto}\text{IX}\left(\mu molg^{-1}\text{Prot}\right)=(0.06077\times {\text{OD}}_{590}-0.01937\times {\text{OD}}_{575}-0.003423\times {\text{OD}}_{628})\times \text{V}1/(\text{V}2\times \text{Cpr})$$

$$\text{Pchlide}\left(\mu molg^{-1}\text{Prot}\right)=(0.03563\times {\text{OD}}_{628}+0.007225\times {\text{OD}}_{590}-0.02955\times {\text{OD}}_{575})\times \text{V}1/(\text{V}2\times \text{Cpr})$$

### Total RNA Extraction and Relative Gene Expression Analysis

Total RNA was extracted using the RNAprep Pure Plant Plus Kit (Tiangen Biotech, Beijing, China) according to the manufacturer’s protocol, which included a genomic DNA elimination step. RNA quality and concentration were checked on a DS-11+ spectrophotometer (DeNovix Inc., Wilmington, DE, United States), and samples were stored at −80°C until further use. cDNA synthesis was performed using a FastKing RT Kit (Tiangen Biotech) according to the manufacturer’s protocol. The tomato actin gene was used as an internal control. The GenBank accession numbers of the sequences used to design the primers are listed in Table 1. Finally, 1 μL of cDNA was used for quantitative real-time PCR (qRT-PCR) analysis in a real-time PCR detection system (QuantStudio 5 Real-Time PCR System; Thermo Fisher Scientific, Waltham, MA, United States). The reaction mixture included the following: 1 μL cDNA template, 10 μL of 2× Talent qPCR PreMix (Tiangen Biotech), 0.4 μL of 50× ROX Reference Dye^Δ^ (Tiangen Biotech), 0.6 μL forward primer, 0.6 μL reverse primer, and RNase-Free ddH_2_O to a final volume of 20 μL. For each sample, there were three wells for the target gene and three wells for the negative control (all components of the reaction mix, except for template cDNA replaced by RNase-free ddH_2_O) on the plate. The two-step amplification procedure consisted of one cycle of pre denaturation at 95°C for 3 min, followed by 40 cycles of denaturation at 95°C for 5 s, annealing and extension at 60°C for 15 s. Each qPCR assay was repeated three times. Quantification analysis was performed using the comparative CT method (Livak and Schmittgen, 2001). The CT value of actin was subtracted from that of the target gene to obtain the ΔCT value. The average CT value of the control sample was subtracted from the ΔCT value to obtain the ΔΔCT value. For each sample, the expression level relative to the control was expressed as 2^–ΔΔCT^.

**TABLE 1:** Primer sequences and GenBank accession numbers of the geranylgeranyl diphosphate synthase (*GGPPS*), *PSY1*, phytoene desaturase (*PDS*), lycopene β-cyclase (*LCY-B*), *CHLH*, *POR*, and *ACTIN* gene sequences.

**Gene symbol**	**Accession number**	**Forward primer 5′–3′**	**Reverse primer 5′–3′**
*GGPPS*	NM_001366706.1	CTGCCTGTGCCTTAGAGATGGTTC	CCTCGTCGAGTTGTGTCATCATCC
*PSY1*	NM_001247883.2	GCTGGAAGGGTGACCGATAAATGG	GTCACGCCTTTCTCTGCCTCATC
*PDS*	NM_001247166.2	CGAGGTCGTCTTCTTTGGGAACTG	CAATCTTCTGGTCGTGGCATGGG
*LCY-B*	NM_001247297.2	GAGTCGTTGGAATCGGTGGTACAG	CAACAGGAGCCGCAGCTAGTG
*CHLH*	XM_004236562.4	GTGCTGGCATGATGGAGAAGAGG	GAGGTTCTGAACGAGGTTGGTTGG
*POR*	NM_001317974.1	TGGACCTCGCCTCTCTTGACAG	CAGCAGCATTAGCAACCAACACG
*ACTIN*	NM_001330119.1	TTGTGTTGGACTCTGGTGATGGTG	GACGGAGAATGGCATGTGGAAGG

### Statistical Analysis

All experiments were performed in triplicate, and data from all experiments were expressed as means ± SE. Data were analyzed by analysis of variance (ANOVA) in SPSS version 23.0 (IBM Corp., Armonk, NY, United States), and treatment means were compared using Tukey’s honest significant difference test at a 0.05 level of probability (*P*-value < 0.05). All figures were prepared using OriginPro 8.5.0 (OriginLab Corporation, Northampton, MA, United States).

## Results

### Effects of Different Concentrations of Exogenous ALA on Soluble Sugar, Soluble Solids Content, and Firmness of Tomato Fruit

**FIGURE 1:**
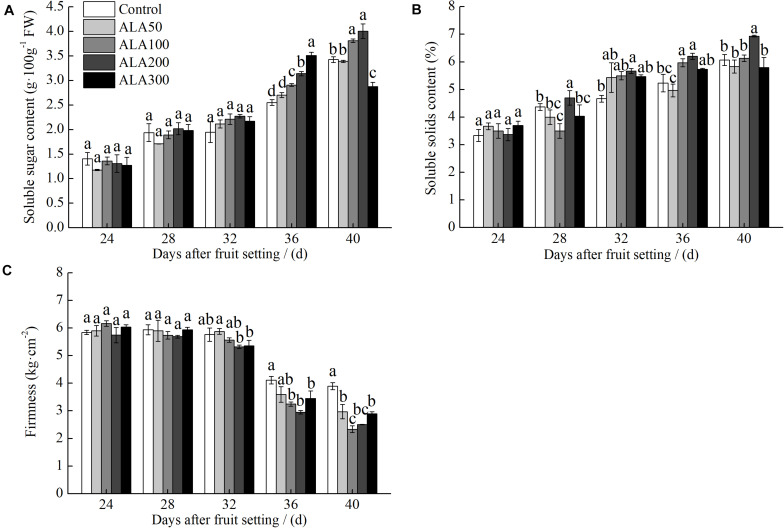
The effects different concentrations of 5-aminolevulinic acid (ALA) on the soluble sugar, soluble solid content, and firmness of tomato fruit. **(A)** Soluble sugar content; **(B)** Soluble solid content; and **(C)** Firmness of tomato fruits at 24th–40th day after fruit setting under different treatments. Vertical bars represent mean ± SE from three independent replicates and the different letters denote significant differences (*p* < 0.05).

As shown in Figure 1A, at 36th day after fruit setting, the soluble sugar content of tomato fruit treated with 200 mg⋅L^–1^ ALA was significantly higher than those of other treatments. When 100 and 200 mg⋅L^–1^ ALA treatments reached maturity (40th day after fruit setting), the soluble sugar content was significantly higher than other treatments. The soluble solids content increased during fruit ripening (Figure 1B), at 36th day after fruit setting, 100 and 200 mg⋅L^–1^ ALA treatments was significantly higher than those in other treatments. At 40th day after fruit setting, 200 mg⋅L^–1^ ALA treatment reached the maximum and was significantly higher than other treatments. As shown in Figure 1C, at 40th day after fruit setting, the fruit firmness of 100–300 mg⋅L^–1^ ALA treatments were significantly lower than that of the control. At 40th day after fruit setting, 100 and 200 mg⋅L^–1^ ALA treatments were significantly lower than the control. Based on the effects of the various ALA concentrations on soluble sugar, soluble solids content and firmness of tomato fruit, we considered the 100 and 200 mg⋅L^–1^ ALA treatments were more effective to increase the content of soluble sugar and soluble solid and reduce fruit firmness. The 100 and 200 mg⋅L^–1^ ALA treatments were employed in the further experiment.

### Protective Effects of ALA on Morphology and Skin Color Parameters of Tomato Fruits

**FIGURE 2:**
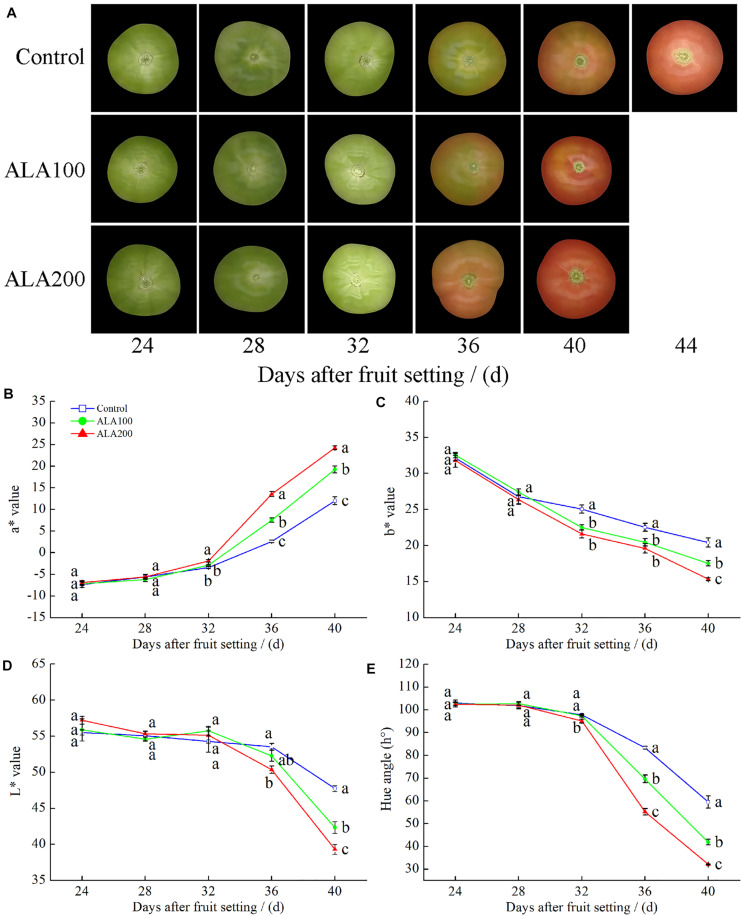
The effects of ALA on morphology and skin color parameters of tomato fruits. **(A)** The fruit morphology; **(B)** the *a*^∗^ value; **(C)** the *b*^∗^ value; **(D)** the lightness; and **(E)** the hue angle of tomato fruits skin at 24th–40th day after fruit setting under different treatments. The data presented represent mean ± SE from three independent replicates and the different letters denote significant differences (*p* < 0.05).

During tomato ripening, i.e., coloration changes from green to red (Figure 2A), ALA treatment resulted in faded green areas on the fruit surface by the 32nd day after fruit setting. On the 36th day after fruit setting, fruits treated with 100 and 200 mg⋅L^–1^ ALA were at the pink stage when the fruit of the control group was at the color break stage. The ALA-treated fruits reached maturity 4 days earlier than the control fruits. The color difference of fruits in each treatment group was compared by measuring the values of *a*^∗^, *b*^∗^, and *L*^∗^ and calculating the hue angle. The *a*^∗^ value, which indicates colors ranging from green to red, gradually increased with tomato fruit development in each treatment (Figure 2B). In contrast, the *b*^∗^ (Figure 2C), *L*^∗^ (Figure 2D), and hue angle (Figure 2E) values showed an overall decreasing trend. In particular, 36–40 days after fruit setting, the *a*^∗^ value of the 200 mg⋅L^–1^ ALA-treated fruit was significantly higher than that of the control. In addition, the values of *b*^∗^ and *L*^∗^, and the hue angle values of the ALA treatment group were significantly lower than those of the control group. Hence, after exogenous ALA treatment, the green fading of the fruit skin was accelerated.

### Exogenous Application of ALA Modulates the Nutrient Quality of Tomato Fruits

As shown in Table 2, the different exogenous ALA concentrations affected the quality indices of tomato fruits. The soluble sugar content of tomato fruit increased continuously, whereas the titratable acid first increased and then decreased. The soluble sugar content of fruit treated with 200 mg⋅L^–1^ ALA was significantly higher than that of the control, 28–40 days after fruit setting. When the fruit of the control group reached maturity (44 days after fruit setting), the soluble sugar content was significantly lower than that of the fruit treated with 200 mg⋅L^–1^ ALA on the 40th day after fruit setting. By day 24–32 after fruit setting, there was no significant effect on titratable acid content among the treatments. The titratable acid content in fruit treated with exogenous ALA decreased by the 36th day after fruit setting, whereas it decreased by the 40th day after fruit setting in the control group. The soluble protein content increased gradually during fruit ripening. Compared with the control group, exogenous ALA treatment significantly increased the soluble protein content in tomato fruit. When the fruits in the control group reached maturity (44 days after fruit setting), there was no significant difference in soluble protein content compared with the ALA treatments by the 40th day after fruit setting. The total free amino acid and soluble protein content showed a similar trend under ALA treatment. Both attained their highest levels under 200 mg⋅L^–1^ ALA treatment on the 40th day after fruit setting. The vitamin C content in tomato fruits increased with fruit development. Compared with the control group, both 100 and 200 mg⋅L^–1^ ALA treatments significantly increased the vitamin C content in tomato fruit. By 36–40 days after fruit setting, 200 mg⋅L^–1^ ALA treatment showed a stronger effect than 100 mg⋅L^–1^ ALA treatment (Table 2).

**TABLE 2:** Effects of ALA on the soluble sugar, titratable acid, soluble protein, total free amino acid, and vitamin C contents of tomato fruits.

**Variables**	**Treatments**	**Days after fruit setting / (day)**					
		**24**	**28**	**32**	**36**	**40**	**44**
Soluble sugar (g⋅100 g^–1^ FW)	Control	2.27 ± 0.07 a	2.54 ± 0.09 b	3.15 ± 0.03 c	3.93 ± 0.09 c	4.35 ± 0.04 b	4.50 ± 0.09 b
	ALA100	2.39 ± 0.05 a	2.89 ± 0.10 a	3.39 ± 0.05 b	4.20 ± 0.05 b	4.57 ± 0.03 b	
	ALA200	2.35 ± 0.06 a	3.05 ± 0.11 a	3.68 ± 0.09 a	4.47 ± 0.05 a	4.92 ± 0.10 a	
Titratable acid (g⋅100 g^–1^ FW)	Control	1.10 ± 0.05 a	1.02 ± 0.04 a	1.19 ± 0.02 a	1.33 ± 0.04 a	1.20 ± 0.01 a	1.04 ± 0.03 b
	ALA100	1.05 ± 0.01 a	0.99 ± 0.03 a	1.15 ± 0.04 a	1.25 ± 0.00 ab	1.10 ± 0.02 ab	
	ALA200	1.09 ± 0.04 a	1.04 ± 0.03 a	1.16 ± 0.02 a	1.19 ± 0.02 b	0.98 ± 0.05 b	
Soluble protein (mg⋅g^–1^ FW)	Control	0.16 ± 0.008 a	0.20 ± 0.008 b	0.49 ± 0.01 c	0.68 ± 0.015 c	0.91 ± 0.03 b	1.15 ± 0.028 a
	ALA100	0.17 ± 0.012 a	0.22 ± 0.007 b	0.60 ± 0.014 b	0.75 ± 0.016 b	1.14 ± 0.015 a	
	ALA200	0.16 ± 0.011 a	0.27 ± 0.011 a	0.70 ± 0.011 a	0.90 ± 0.014 a	1.22 ± 0.025 a	
Total free amino acid (mg⋅100 g^–1^ FW)	Control	12.44 ± 0.25 a	12.66 ± 0.56 b	14.64 ± 0.20 c	19.36 ± 0.42 b	21.56 ± 0.45 c	23.43 ± 0.31 b
	ALA100	12.71 ± 0.15 a	14.86 ± 0.38 a	17.11 ± 0.33 b	20.35 ± 0.44 b	23.10 ± 0.36 b	
	ALA200	12.49 ± 0.67 a	14.91 ± 0.29 a	18.21 ± 0.34 a	21.89 ± 0.24 a	25.74 ± 0.57 a	
Vitamin C (mg⋅100 g^–1^ FW)	Control	5.54 ± 0.26 a	6.34 ± 0.32 b	7.10 ± 0.26 b	7.94 ± 0.29 b	9.95 ± 0.44 c	11.37 ± 0.25 b
	ALA100	5.82 ± 0.47 a	7.11 ± 0.23 ab	8.07 ± 0.25 a	8.71 ± 0.34 b	11.74 ± 0.36 b	
	ALA200	6.12 ± 0.34 a	7.58 ± 0.05 a	8.37 ± 0.12 a	10.44 ± 0.28 a	13.02 ± 0.13 a	

*The 44^*th*^ day after fruit setting data of the control group and the 40^*th*^ day after fruit setting data of other treatments were analyzed by ANOVA to separate its significance. Each value represents mean ± SE from three independent replicates and the different letters denote significant differences (*p* < 0.05).*

The 44th day after fruit setting data of the control group and the 40th day after fruit setting data of other treatments were analyzed by ANOVA to separate its significance. Each value represents mean ± SE from three independent replicates and the different letters denote significant differences (*p* < 0.05).

### Effects of Exogenous ALA on Amino Acid Components of Tomato Fruit

**FIGURE 3:**
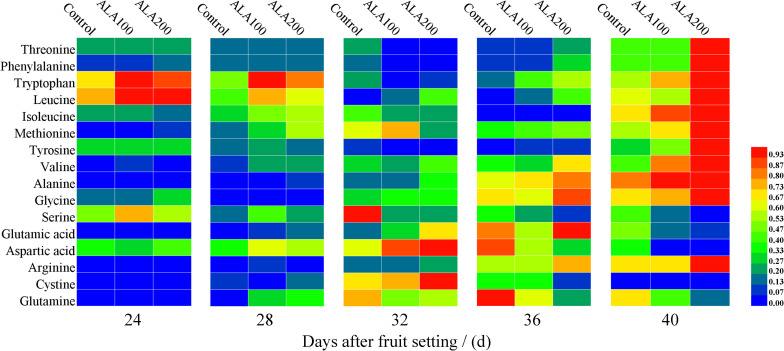
Heat map of the effects of ALA on amino acid components in tomato fruit. Min-Max normalization method was used to standardize the data (Mohamad and Usman, 2013). The color block represents the relative value of amino acid components in the corresponding position.

As shown in Figure 3, the different exogenous ALA treatments affected the amino acid components of tomato fruits. The contents of threonine, phenylalanine, tryptophan, leucine, isoleucine, methionine, tyrosine, valine, alanine, glycine, and arginine of fruit treated with 200 mg⋅L^–1^ ALA was significantly higher than that of the control, at 40 days after fruit setting. During 24–32 day after fruit setting, there was no significant effect on alanine content among the treatments. However, the alanine content treated with 200 mg⋅L^–1^ ALA increased by the 36th day after fruit setting, it was significantly higher than that of the control. The glutamic acid content of each treatment reached the maximum by the 36th days after fruit setting, and 200 mg⋅L^–1^ ALA treatment was significantly higher than that of the control. Then, glutamate content decreased markedly, and ALA treatment was significantly lower than the control. Similarly, glutamine and glutamic acid have similar trends. On the 40th day after fruit setting, ALA treatment was significantly lower than the control, and the effect of 200 mg⋅L^–1^ ALA treatment was more significant. Data of amino acid component contents were revealed in Supplementary Table 1.

### Ameliorative Role of ALA in the Lycopene Synthetic and Metabolic Pathways

**FIGURE 4:**
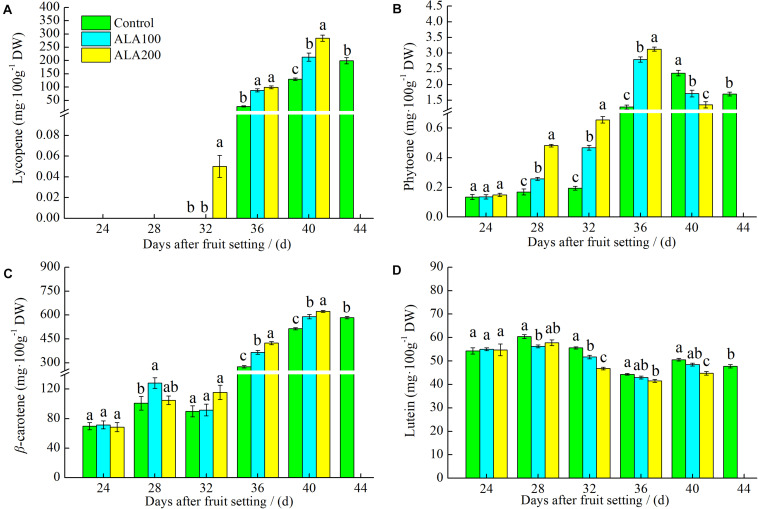
The effects of ALA on the intermediates in lycopene synthesis and metabolic pathway. **(A)** Lycopene content; **(B)** the phytoene content; **(C)** the β-carotene content; and **(D)** the lutein content of tomato fruits at 24th–44th day after fruit setting under different treatments. The 44th day after fruit setting data of the control group and the 40th day after fruit setting data of other treatments were analyzed by analyzed by analysis of variance (ANOVA) to separate its significance. Vertical bars represent mean ± SE from three independent replicates and the different letters denote significant differences (*p* < 0.05).

We determined the lycopene, phytoene, β-carotene, and lutein contents of tomato fruits 24–44 days after fruit setting. The lycopene content increased as the fruits ripened (Figure 4A). Lycopene was not detected in either control or ALA-treated fruits 24–28 days after fruit setting. On the 32nd day after fruit setting, a small amount of lycopene was detected but only in the 200 mg⋅L^–1^ ALA-treated fruit. By day 36–40 after fruit setting, the lycopene content of ALA-treated fruit was significantly higher than that of the control fruit, and lycopene levels were highest after 200 mg⋅L^–1^ ALA treatment 40 days after fruit setting. However, when the control group reached maturity (44 days after fruit setting), the lycopene content was significantly lower than that of mature fruits treated with 200 mg⋅L^–1^ ALA (40 days after fruit setting). Phytoene content first increased and then decreased with fruit ripening (Figure 4B). Phytoene content in fruits treated with ALA was significantly higher than that of the control by 28–36 days after fruit setting but decreased on the 40th day after fruit setting. During the ripening process, the β-carotene content continuously increased (Figure 4C). The β-carotene content was higher in ALA-treated fruits than in the controls. The lutein content initially decreased and then increased during the ripening process of tomato fruit (Figure 4D). However, it remained at a similarly low level in ALA-treated fruits. On the 32nd, 36th, and 40th day after fruit setting, the lutein content of the 200 mg⋅L^–1^ ALA-treated fruit was significantly lower than that of the controls.

### Relative Expression of Key Genes Involved in the Lycopene Synthetic and Metabolic Pathways

**FIGURE 5:**
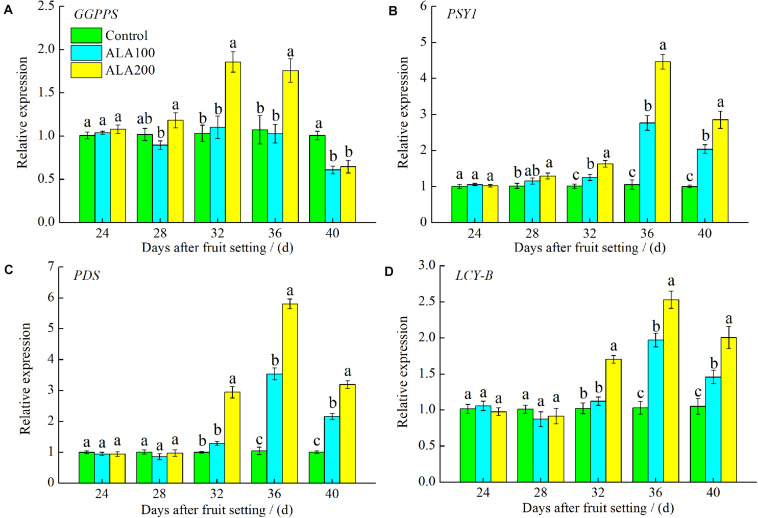
Relative expression levels of key genes involved in lycopene synthesis and metabolism pathway. **(A)** Relative expression of *GGPPS*, encoding geranylgeranyl diphosphate synthase; **(B)** relative expression of *PSY1*, encoding phytoene synthase; **(C)** relative expression of *PDS*, encoding phytoene desaturase; and **(D)** relative expression of *LCY-B*, encoding lycopene β-cyclase. Vertical bars represent mean ± SE from three independent replicates and the different letters denote significant differences (*p* < 0.05). The control group was used as reference at each sampling time point.

The relative expression levels of the five key genes involved in the lycopene synthetic and metabolic pathways are shown in Figure 5. Compared with control fruits, the expression level of *GGPPS* increased in the 200 mg⋅L^–1^ ALA treatment and reached its highest level 32 days after fruit setting (Figure 5A). On the 32nd and 36th day after fruit setting, the expression level of *GGPPS* in 200 mg⋅L^–1^ ALA-treated fruit was 0.81- and 0.64-fold higher than that of the control. As shown in Figure 5B, the expression levels of *PSY1* in ALA-treated fruit increased gradually 28–36 days after fruit setting, reaching a maximum 36 days after fruit setting. The expression level of *PSY1* decreased slightly on the 40th day compared with the 36th day after fruit setting. Moreover, 200 mg⋅L^–1^ ALA significantly upregulated *PSY1* expression compared with that of 100 mg⋅L^–1^ ALA and the control group 32–40 days after fruit setting. Compared with the control, both 100 and 200 mg⋅L^–1^ ALA treatments significantly upregulated *PDS* and *LCY-B* in tomato fruits. By 32–40 days after fruit setting, the expression levels of *PDS* and *LCY-B* in fruits treated with 200 mg⋅L^–1^ ALA were significantly higher than those in the 100 mg⋅L^–1^ ALA treatment and control groups (Figures 5C,D).

### Effects of ALA on Endogenous Metabolic Derivatives of the ALA Metabolic Pathway

**FIGURE 6:**
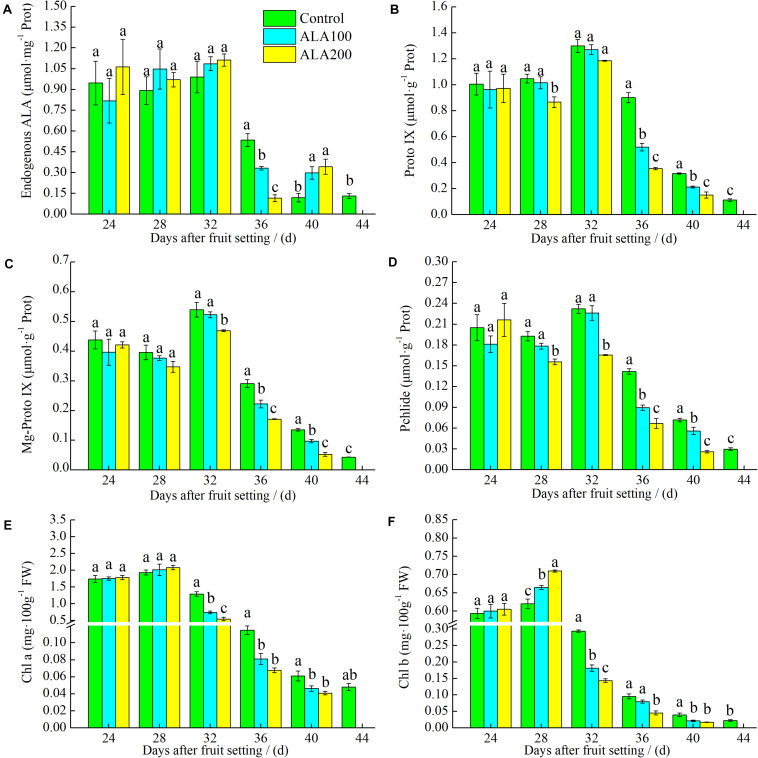
The effects of ALA on endogenous derivatives of ALA metabolic pathway. **(A)** Endogenous ALA content; **(B)** Proto? content; **(C)** Mg-Proto? content; **(D)** Pchlide content; **(E)** Chl *a* content; and **(F)** Chl *b* content of tomato fruits at 24th–44th day after fruit setting. The 44th day after fruit setting data of the control group and the 40th day after fruit setting data of other treatments were analyzed by ANOVA to separate its significance. Vertical bars represent mean ± SE from three independent replicates and the different letters denote significant differences (*p* < 0.05).

As shown in Figure 6A, 24–32 days after fruit setting, the endogenous ALA content in ALA-treated fruits was no significant difference compared with the control. However, on the 36th day after fruit setting, endogenous ALA content in tomato fruit treated with ALA decreased significantly. Subsequently, endogenous ALA content increased with increasing treatment concentration. Under exogenous ALA treatments, the Proto IX, Mg-Proto IX, and Pchlide contents of the fruits initially increased and then later decreased (Figures 6B–D). On the 32nd day after fruit setting, they reached their highest levels in all treatments. In addition, their values in fruits under 200 mg L^–1^ ALA treatment were significantly lower than those of the control group on the 40th day after fruit setting. As shown in Figures 6E,F, on the 28th day after fruit setting, the chlorophyll a content in fruits was slightly increased by exogenous ALA, but there was no significant difference compared with the control. However, the chlorophyll *b* content was significantly higher than that of the control. Application of exogenous ALA inhibited the total content of chlorophyll 32–40 days after fruit setting due to the reduction of both chlorophyll *a* and *b* contents. In addition, for 200 mg⋅L^–1^ ALA treatment, chlorophyll *a* and *b* contents were significantly decreased compared with the control.

### Relative Expression Levels of Key Genes Involved in the ALA Metabolic Pathway

**FIGURE 7:**
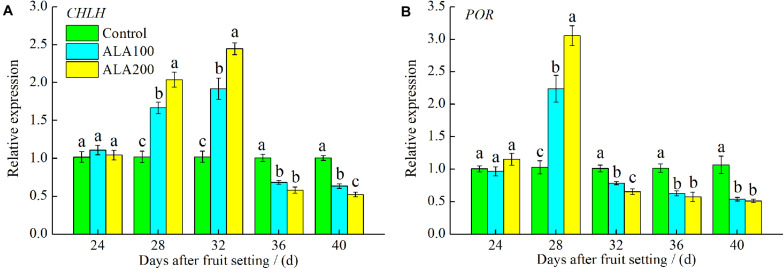
Relative expression of genes involved in ALA metabolic pathway. **(A)** Relative expression of *CHLH*, encoding Mg-chelatase; **(B)** relative expression of *POR*, encoding protochlorophyllide oxidoreductase. Vertical bars represent mean ± SE from three independent replicates and the different letters denote significant differences (*p* < 0.05). The control group was used as reference at each sampling time point.

The relative expression levels of genes involved in the ALA metabolic pathway during tomato fruit ripening were determined and are shown in Figure 7. By 28–32 days after fruit setting, the expression level of *CHLH* was significantly increased by exogenous ALA. In addition, the expression level of *CHLH* showed a decline by 36–40 days after fruit setting (Figure 7A). The expression level of *POR* was significantly increased by ALA treatment by the 28th day after fruit setting but was subsequently inhibited (Figure 7B).

## Discussion

As a plant growth regulator, ALA has the great potential for use in agricultural production (Akram and Ashraf, 2013), especially in the field of improving fruit color. For example, the application of exogenous ALA to apple fruits significantly increased the anthocyanin content in the fruit skin, thereby improving fruit color (Xie et al., 2013; Feng et al., 2016). Moreover, ALA significantly improved the fruit coloring of “Feizixiao” lychee at harvest (Feng et al., 2015). Thus, in the production of red fruits, exogenous ALA seems to be widely used to improve fruit color (Zheng et al., 2018). However, little information is available on regulatory mechanisms behind ALA-induced tomato fruit coloration. In the present study, ALA (100 and 200 mg L^–1^) improved the color of tomato fruits and accelerated the ripening process (including color break, pink, and red stages), and the harvesting process occurred 4 days earlier than the control. Harvesting early reduces capital and energy inputs (Xie et al., 2017) and allows for early marketing to obtain economic benefits (Wang et al., 2012).

The color of tomato fruit is mainly related to its pigments, including chlorophyll, carotenoids, and anthocyanins, and their relative proportions (Borghesi et al., 2016). And coloration is mainly attributable to lycopene accumulation in red tomato fruits (Ip et al., 2013). In the present study, ALA application promoted the accumulation of carotenoids in tomato fruits. In particular, on the 40th day after fruit setting, the lycopene content of tomato fruits treated with 200 mg⋅L^–1^ ALA was significantly higher than that of the controls, indicating that exogenous ALA promoted the synthesis and accumulation of lycopene, which promoted the coloring of tomato fruit. However, the molecular mechanism of ALA on lycopene accumulation in tomato fruits have not been elucidated. In fruits of higher plants, lycopene biosynthesis and metabolism are intricate processes involving a series of physiological and biochemical reactions and various genes (such as *GGPPS*, *PSY*, *PDS*, and *LCY-B*) (Li et al., 2020). PSY is a key rate-limiting enzyme of lycopene biosynthesis in ripening tomato fruits (Rodríguez-Villalón et al., 2010; Stuart et al., 2011). The absence of *PSY1* in tomato fruits results in a yellow-flesh phenotype (Kang et al., 2014). Loss of *PSY1* gene function by gene silencing and CRISPR/Cas9 techniques resulted in yellow tomato fruits, confirming the function of the *PSY1* gene (D’Ambrosio et al., 2018). In the present study, the overall expression of genes related to lycopene synthesis was upregulated by exogenous ALA in tomato fruits. Relative expression levels of *GGPPS*, *PSY1*, and *PDS* 32–36 days after fruit setting were increased by the application of 200 mg L^–1^ ALA, thus promoting lycopene accumulation. Furthermore, when the tomato fruit is fully ripe, all-*trans-*lycopene is converted to β- and α-carotene, catalyzed by lycopene β-cyclase (LCY-B) and lycopene ε-cyclase (LCY-E), respectively (Cunningham et al., 1994; Ronen et al., 1999). β- and α-carotene are precursors of zeaxanthin and lutein, respectively (Fraser et al., 2002). Overexpression of *NtLCYB* results in tomato fruit with high β-carotene content (Ralley et al., 2016), indicate *LCY-B* plays an important role in lycopene metabolism. In the present study, ALA significantly upregulated the expression of *LCY-B*, thereby increasing the content of β-carotene. The expression patterns of the genes involved in carotenoids metabolism after ALA treatment were consistent with carotenoids accumulation, these results further indicate that exogenous ALA can improve the external color of tomato fruit. ALA secreted by *Rhodopseudomonas* sp. inoculant in the cultivation substrate could increase tomato fruit lycopene content (Lee et al., 2008). Therefore, the positive role of ALA in lycopene accumulation in fruits is probably caused by ALA-induced gene expression during lycopene synthesis.

With the maturity of tomato fruit, the color of fruit changed significantly, mainly in the carotenoids content increase and the degradation of chlorophyll (Borghesi et al., 2016). As a precursor of chlorophyll synthesis, the ALA metabolic pathway and chlorophyll degradation are closely related to fruit ripening (Senge et al., 2014). For example, during tomato fruit ripening, it was found that *glutamate-1-semialdehyde aminotransferase* (*GAST*) was downregulated at the transcription level (Kyriacou et al., 1996). Meanwhile, 5-aminolevulinic acid dehydratase (ALAD), a key enzyme in ALA metabolism, was significantly downregulated at the translation and enzymatic activity levels, and the content of chlorophyll in the fruit decreased (Polking et al., 1999). These results indicate that chlorophyll synthesis is inhibited during tomato ripening. In the present study showed that ALA application increased chlorophyll synthesis (including Proto IX, Mg-Proto IX, and Pchlide) in the early stage of fruit development, which subsequently decreased rapidly 36 days after fruit setting. In *Brassica napus*, the relative expression of Glutamyl–tRNA synthetase (*GLUTS*), a gene in the ALA biosynthetic pathway, was upregulated by exogenous ALA, and Proto IX, Mg-Proto IX, and Pchlide levels were increased (Xiong et al., 2018). Moreover, spraying ALA on cucumber leaves significantly increased their chlorophyll content (Wu et al., 2018). Similar results were found in the present study; the contents of chlorophyll *a* and *b* increased during early fruit development after ALA application, and subsequently, chlorophyll synthesis was significantly inhibited during the late stage of fruit development. In addition, during the early stage of fruit maturity (24–32 days after fruit setting), the endogenous ALA maintained a relative stable level in tomato fruit. When fruits grew into breaker stage (36th day after fruit setting), endogenous ALA significantly declined by exogenous ALA application. Then, it was accumulated when the fruits were in pink stage (40th day after fruit setting). In the early growth stage, the fruit is a photosynthetic tissue (Hiratsuka et al., 2015). We believe that exogenous ALA promotes chlorophyll synthesis. However, application of ALA advanced the breaker stage of fruit and promoted the synthesis of carotenoid pigments. Then, chlorophyll synthesis was inhibited in the late stage of fruit development, which resulted in the inhibition of ALA metabolism in the fruit. These results suggested that the metabolism of ALA and biosynthesis of carotenoid in tomato fruit might have some interaction regulative mechanism, which should be deeply explored in further studies. In higher plants, MCH and POR are the main catalytic enzymes involved in chlorophyll biosynthesis (Nickelsen et al., 2011; Akram and Ashraf, 2013; Sobotka, 2014). MCH consists of three subunits, CHLH, CHLI, and CHLD, in higher plants; among these, CHLH is primarily responsible for the catalytic action of MCH (Richter and Grimm, 2013). Introduction of the HEMA-RNA-interference (RNAi) gene into tobacco (*Nicotiana tabacum* L.) resulted in a decline in MCH activity and caused a decrease in chlorophyll content (Hedtke et al., 2007). In the present study, 28–32 days after fruit setting, *CHLH* expression was significantly increased by exogenous ALA. However, the gene expression of *CHLH* was downregulated 36–40 days after fruit setting. These results are consistent with the change in the Mg-Proto IX content after ALA treatment. Moreover, ALA treatment significantly promoted *POR* gene expression on the 28th day after fruit setting. Subsequently, *POR* expression was downregulated immediately before the tomato fruit reached the color break stage (32 days after fruit setting). The downregulation of *POR* resulted in the accumulation of Pchlide, which led to a decline in ALA metabolism since there is feedback regulation between ALA and Pchlide synthesis (Richter et al., 2010). These results further indicated that exogenous ALA treatment significantly promoted chlorophyll synthesis at the early stage of fruit development and that feedback inhibition eventually led to the suppression of chlorophyll biosynthesis during the late period of fruit growth. In addition, some studies have shown that inhibition of lycopene cyclase results in the accumulation of a large amount of chlorophyll synthesis precursors in plant tissues, indicating an interaction between the lycopene synthetic pathway and the ALA metabolic pathway (La Rocca et al., 2007). In the present study, the accumulation and synthesis of lycopene in tomato fruits treated with exogenous ALA were higher than those in the control fruits. Possible reasons for this phenomenon are as follows. First, during the fruit coloration period, chlorophyll began to break down, and lycopene synthesis was dominant. It is possible that feedback regulation of the ALA downstream metabolic pathway might relate to lycopene synthesis (Richter et al., 2010). Second, we speculate that ALA promotes lycopene synthesis by activating endogenous ethylene synthesis (Rupasinghe, 1995; Klee and Giovannoni, 2011). However, the specific mechanism has not been elucidated and therefore requires further investigation.

The results showed that application of exogenous ALA could not only improve the external quality of fruit, but also increase the internal quality (Xie et al., 2013). For example, the content and proportion of sugars and acids are important factors in fruit internal flavor quality (Hecke et al., 2006; Kafkas et al., 2007). Exogenous ALA could promote the accumulation of photosynthetic products, such as sugar and starch, thus promoting crop quality (Hotta et al., 1997). In grapefruit, the soluble solid content increased by 2.7%, and the organic acid content decreased significantly with the application of 100 mg⋅L^–1^ ALA (Watanabe et al., 2006). In the present study, we applied 200 mg⋅L^–1^ ALA on the 24th day after fruit setting (the mature green stage); this increased the soluble sugar content of the tomato fruits, decreased the titratable acid content between days 36 and 40 after fruit setting, and increased the sugar-acid ratio, resulting in sweeter fruit. It has been suggested that exogenous ALA increases the fructan (polyfructosylsucrose) content, suggesting that ALA is related to the transfer and storage of carbohydrates, as well as to the formation of polysaccharides in higher plants (Bingshan et al., 1998). Moreover, the sugar and acid contents in fruit treated with 200 mg⋅L^–1^ ALA at its mature stage (40th day after fruit setting) were consistent with the control fruit levels at maturity (44 days after fruit setting). Because proteins are composed of different amino acids, their content can be determined by the amounts, proportions, and availability of their amino acids (Gressler et al., 2010). The results of the present study showed that exogenous ALA significantly increased the contents of soluble protein and total free amino acids in the tomato fruits, and they changed in the same way. Recently, similar results were observed when rhizospheric irrigation with 160 mg⋅L^–1^ ALA significantly increased the soluble protein content in apple fruit (Zheng et al., 2017). The free amino acid content of *Brassica napus* was further increased by foliar application of ALA (Naeem et al., 2010). Vitamin C plays an important role in protecting human health by effectively halting tumor development, and vitamin C plays an important role in the growth and development of plants (Drisko et al., 2018). Ye et al. (2017) found that when 200 or 400 mg⋅L^–1^ ALA solution was sprayed to the fruit surface before the peach fruit reached maturity, and the vitamin C content in fruit flesh was significantly higher than that in control. Exogenous application of ALA to lychee peel, the vitamin C content of the fruits increased by 9–13% at harvest (Feng et al., 2015). In the present study, at 36th–40th day after fruit setting, application of 100 and 200 mg⋅L^–1^ ALA solution to the tomato fruit surface significantly increased the vitamin C content compared with the control. This result is likely related to the participation of vitamin C in plant respiration, ALA may weaken the respiration of plants by promoting the photosynthesis of tomato fruit, leading to increase in vitamin C content in fruit (Ye et al., 2017).

Amino acid is an important parameter reflecting the flavor and nutritional value of tomato fruit (Goff and Klee, 2006; Sorrequieta et al., 2010). It cannot only be used to regulate protein synthesis, maintain nitrogen balance, and other related physiological functions, but also closely related to human taste perception (Salvioli et al., 2012). Some amino acids, such as aspartic acid and glutamic acid, may contribute to sourness, while alanine, glycine and serine more contribute to sweetness (Kader, 2008). In the present study, the contents of threonine, phenylalanine, tryptophan, leucine, isoleucine, methionine, tyrosine, valine, alanine, glycine, and arginine of fruit treated with 200 mg⋅L^–1^ ALA was significantly higher than that of the control, 40 days after fruit setting. Glutamic acid, as the highest content of amino acid in tomato fruit, has more contribution to fruit flavor quality (Cheng et al., 2019). In the present study, after exogenous 200 mg⋅L^–1^ ALA treatments, the content of glutamic acid was significantly higher than that of the control on the 36th day after fruit setting. This is because glutamic acid is the source of ALA synthesis (Czarnecki and Grimm, 2012), while the synthesis of endogenous ALA in fruit treated with 200 mg⋅L^–1^ ALA was inhibited on the 36th day after fruit setting. Eventually leading to the accumulation of glutamic acid. These results further indicate that exogenous ALA can improve the flavor quality of tomato fruit. In summary, exogenous ALA application to mature green stage tomato fruits could promote coloration and ripening and improve internal qualities.

## Conclusion

Exogenous ALA, the optimal effect on 200 mg L^–1^, had positive effects on tomato fruit coloration and improved carotenoid biosynthesis by upregulating the expression of genes related to lycopene synthesis and metabolism (including *GGPPS*, *PSY1*, *PDS*, and *LCY-B*). Moreover, the chlorophyll synthesis pathway was inhibited mainly by downregulating the related genes (*CHLH* and *POR*) simultaneously. In addition, the application of exogenous ALA increased the contents of soluble sugar, soluble protein, vitamin C, and total free amino acids as well as 11 kinds of amino acid components in tomato fruits and reduced the content of titratable acid, thus improving fruit quality. Our study showed that tomato fruits treated with exogenous ALA reached maturity 4 days earlier than the controls. In summary, the application of an appropriate concentration of ALA (200 mg⋅L^–1^) during ripening enhances fruit nutritional qualities, coloration and promotes fruit maturation in tomato.

## Data Availability Statement

The original contributions presented in the study are included in the article/Supplementary Material, further inquiries can be directed to the corresponding authors.

## Author Contributions

YW and JY conceived and designed the research. JW, JZ, and JLi conducted the experiments. JW, JZ, and YW analyzed the data and prepared the figures and illustrations. JW wrote the manuscript. MD, BA, ZT, JLy, XX, LH, and JX read the manuscript and made valuable inputs. All authors read and approved the submission of the manuscript.

## Conflict of Interest

The authors declare that the research was conducted in the absence of any commercial or financial relationships that could be construed as a potential conflict of interest.
