# Supplementary material for: Exogenous Application of 5-Aminolevulinic Acid Promotes Coloration and Improves the Quality of Tomato Fruit by Regulating Carotenoid Metabolism
Source: Front Plant Sci. 2021 Jun 9;12:683868. doi: 10.3389/fpls.2021.683868 (PMC8243651; doi:10.3389/fpls.2021.683868)
Supplement: Supplementary file 1 [file Table_1.docx]

**Table S1 Contents of free amino acid components** (mg·kg^-1^ DW)

| Categories of free amino acids | 24 day | | | 28 day | | | 32 day | | | 36 day | | | 40 day | | |
| --- | --- | --- | --- | --- | --- | --- | --- | --- | --- | --- | --- | --- | --- | --- | --- |
|  | CK | ALA100 | ALA200 | CK | ALA100 | ALA200 | CK | ALA100 | ALA200 | CK | ALA100 | ALA200 | CK | ALA100 | ALA200 |
| Threonine | 69.81 | 65.64 | 69.30 | 58.14 | 61.48 | 56.80 | 78.74 | 21.77 | 36.22 | 48.83 | 48.12 | 82.08 | 122.29 | 123.34 | 246.92 |
| Phenylalanine | 45.52 | 41.83 | 49.21 | 56.36 | 66.83 | 53.38 | 51.89 | 20.56 | 28.10 | 42.34 | 47.81 | 84.74 | 127.34 | 129.69 | 253.58 |
| Tryptophan | 140.78 | 164.98 | 160.41 | 128.28 | 184.14 | 162.07 | 101.46 | 72.70 | 85.96 | 89.49 | 121.86 | 139.17 | 153.87 | 176.60 | 213.26 |
| Leucine | 75.45 | 85.33 | 82.77 | 64.61 | 81.17 | 74.44 | 42.52 | 50.13 | 65.04 | 43.70 | 50.53 | 64.32 | 73.13 | 71.88 | 92.78 |
| Isoleucine | 53.17 | 55.00 | 48.90 | 62.33 | 80.00 | 90.57 | 72.43 | 58.22 | 53.80 | 37.87 | 33.89 | 37.52 | 101.65 | 122.73 | 130.35 |
| Methionine | 3.14 | 3.36 | 3.50 | 4.14 | 4.86 | 6.00 | 6.63 | 7.04 | 4.21 | 5.25 | 5.37 | 5.82 | 6.05 | 6.79 | 8.46 |
| Tyrosine | 93.61 | 91.64 | 99.49 | 76.94 | 99.97 | 66.15 | 56.42 | 21.54 | 30.88 | 28.80 | 45.15 | 55.75 | 103.39 | 175.64 | 314.73 |
| Valine | 10.29 | 12.33 | 11.39 | 12.79 | 17.33 | 17.22 | 18.31 | 17.22 | 22.22 | 20.28 | 19.00 | 30.04 | 22.62 | 32.97 | 37.47 |
| Alanine | 268.35 | 260.33 | 282.12 | 274.60 | 285.33 | 298.79 | 352.01 | 341.65 | 435.35 | 601.01 | 619.21 | 705.93 | 706.48 | 755.27 | 774.80 |
| Glycine | 2.42 | 2.68 | 3.30 | 1.42 | 1.76 | 1.63 | 3.72 | 4.28 | 4.35 | 6.76 | 6.37 | 8.41 | 6.95 | 7.42 | 9.37 |
| Serine | 45.21 | 53.25 | 48.21 | 36.88 | 44.91 | 38.21 | 67.55 | 37.80 | 38.98 | 43.43 | 39.77 | 33.84 | 44.98 | 35.24 | 29.76 |
| Glutamic acid | 4969.20 | 5154.82 | 4876.33 | 5434.20 | 5711.49 | 6562.99 | 6936.61 | 8520.56 | 12712.49 | 14020.89 | 11160.68 | 16283.41 | 10336.73 | 6522.51 | 5696.97 |
| Aspartic acid | 1284.30 | 1197.00 | 1446.40 | 1450.96 | 1863.67 | 1779.73 | 1845.02 | 2292.50 | 2504.67 | 2345.55 | 1743.85 | 1266.72 | 1452.31 | 774.98 | 785.69 |
| Arginine | 37.08 | 40.40 | 33.18 | 48.75 | 52.90 | 45.68 | 76.15 | 83.77 | 93.62 | 187.10 | 201.09 | 248.56 | 223.33 | 227.62 | 314.34 |
| Cystine | 4.29 | 4.01 | 4.49 | 5.13 | 4.84 | 6.15 | 16.04 | 17.14 | 22.18 | 10.81 | 10.59 | 5.19 | 4.52 | 4.42 | 3.57 |
| Glutamine | 88.52 | 92.89 | 98.13 | 101.02 | 184.55 | 206.47 | 340.76 | 248.73 | 269.92 | 415.83 | 290.13 | 155.98 | 308.21 | 223.36 | 146.11 |
